# The American Medical Association at Atlantic City

**Published:** 1900-07

**Authors:** 


**A Monthly Review of Medicine and Surgery.**

*EDITOR:*

WILLIAM WARREN POTTER, M. D.

All communications, whether of a literary or business nature, books for review and exchanges should be addressed to the editor: 284 FRANKLIN STREET, BUFFALO, N. Y.

VOL. XXXIX.—LV. JULY, 1900. NO. 12.

### THE AMERICAN MEDICAL ASSOCIATION AT ATLANTIC CITY.

THE month of June is almost universally acknowledged to be the most delightful of the year, hence it is a fortunate time for the meeting of the greatest medical organisation of the land. When, added to this, it chooses its place on the sea coast to hold its annual convention, especially at so charming a point as Atlantic City, there is little left to be desired as to time or place for such a gathering of distinguished men and women from all parts of the country.

The meeting held June 5-8, 1900, will ever be conspicuous for size, quality of attendance and work done, and for good feeling engendered. The weather was delightful the whole time, and every body seemed contented and happy. The sections were well managed, and the attendance at each was more than ordinarily good, while the work in some was of a conspicuous character. The weekly journals have chronicled this and we shall content ourselves with this general allusion to the general fact.

The registration was over 2,000 which classes the meeting among the largest ever held. This is an indication of the growth of the republic and of the increasing interest manifested in the advancement of medicine along the lines of scientific research. In the distribution of the offices there were no manifestations of unfairness, hence there are no heartburnings left behind. The two most conspicuous candidates for the presidency were men of acknowledged ability and both were well equipped for the task. Dr. Wheaton, of St. Paul, is one of the most eminent surgeons of the Northwest and every way worthy of the place for which his friends urged him with a spirit of manliness that deserved success. That he was not chosen is no reflection on the candidate or his followers. The spirit in which the canvass was conducted augurs well for his success next time.

Dr. Charles A. L. Reed, of Cincinnati, the president-elect, came to his office with a prestige vouchsafed to few, and perhaps surpassed by none. He is in the very prime of a well-rounded manhood, a scholar of ripe culture, natural and graceful speaker, and as an acquaintance with the profession in the Western hemisphere second to no member of the association. That his administration will result in great advantage to the cause of medical science admits of no reasonable doubt. By a sad *contre-temps* Dr. Reed was summoned home on account of a grievous family affliction, just at the moment when his name was offered to the convention as its next presiding officer. The president at once courteously introduced Dr. Reed and the latter accepted the office in a few eloquent words that well-bespoke the character of the man, who had but just received at the hands of his colleagues the highest indication of the esteem and regard of a great profession.

That one extreme follows another was exemplified again in the selection of the next place of meeting—St. Paul, June 4-7, 1901. The remoteness from the center of the city chosen will perhaps prevent a great attendance, but will be a good meeting and warm hearts will greet the visitors.

As many of the members were returning home they were arrested on their way through Philadelphia by invitations to partake of hospitalities, both public and private, that further exemplified the proverbial courtesy that reigns in the city of brotherly love. The Medical Club, on Saturday evening, June 9th, tendered a reception to Drs. Abraham Jacobi, of New York; Alonzo Garcelon, of Maine, Frank Billings and George H. Simmons, of Illinois, and J. W. Bodine, of Kentucky. The Bellevue, where the reception was held, was crowded to overflowing and, between the supper, the speeches, and the geniality of the hosts, good fellowship was at large until midnight.

---

Dr. F. X. Dercum, professor of neurology at Jefferson Medical College; Dr. Frank Woodbury, professor of laryngology at the Philadelphia Polyclinic; Dr. Judson Daland, editor of the *International Clinics*, and other prominent Philadelphia physicians, entertained many of the visitors at breakfasts and dinners on Saturday. Such courtesies are always appreciated and betoken a spirit of friendliness that serves to unite professional men in a closer bonds of union and friendship.

---

Buffalo was well represented at the meeting. Among those present were Drs. Roswell Park, Herman Mynter, Charles G. Stockton, Allen A. Jones, Julius Ullman, Ernest Wende, S. A. Dunham, A. A. Hubbell, Lorenzo Burrows, A. L. Benedict, Delancey Rochester and J. W. Grosvenor.

---

The Medical Editors' dinner, on Monday evening, June 4th, at The Dennis, which at 3 o'clock in the afternoon threatened a failure, turned out a great success. The number present was large, the speeches were good, and the treasury showed a balance on hand.

---

The section on pediatrics gave a dinner as usual, but this year it was in part a compliment to Dr. Abraham Jacobi, to whom a loving cup was presented. Dr. F. X. Dercum, the eminent Philadephia neurologist, was present by invitation at this dinner, and made one of the happiest of speeches, that did credit to himself as well as the guest of honor.

---

PENNSYLVANIA is following the current of opinion in regard to the out-door treatment of consumption.

Dr. J. T. Rothrock, commissioner of forestry in that state, is strongly in favor of throwing open to consumptives the forest lands owned by the state. He says these reservations are the property of the people, and are to be used wholly in the public interest. The relation of the high woodlands of Pennsylvania to public health was distinctly recognised when the act creating the forestry commission was drawn up. It was provided that the president of the state board of health should be ex-officio a member of the commission which located the forestry reservations, so that these reservations were from the start pledged to the maintenance of public health, so far as they could be serviceable.

In a recently reported interview Dr. Rothrock is reported as saying that it has been suggested, not without careful consideration, that it would probably prove a wise policy if the state were to erect cheap frame buildings, some large enough for a family, some suitable for a few friends, which would provide simply shelter from wind and rain and a safe place for a cheerful fire; in other words, furnish all of the open air advantages of a camp, without its unnecessary exposure, and that these booths should be so located that the reservation guard would be on hand to prevent danger from fire or dissipation, which would defeat the healthful influences of the plan. These buildings could be rented for a nominal sum for a month or for a season, from May until December. Those occupying them could do their own cooking and live as cheaply or as expensively as they wished. The expense to the state would be almost nothing, because such buildings could be erected as needed on a few days' notice, and the rental would soon pay first cost.

This appears to be a feasible and economical plan for state control of incipient consumptives and we hope it will be given a trial.

